# Emergence of Methicillin-Resistant *Staphylococcus aureus* of Animal Origin in Humans

**DOI:** 10.3201/eid1312.070384

**Published:** 2007-12

**Authors:** Inge van Loo, Xander Huijsdens, Edine Tiemersma, Albert de Neeling, Nienke van de Sande-Bruinsma, Desiree Beaujean, Andreas Voss, Jan Kluytmans

**Affiliations:** * Elisabeth Hospital, Tilburg, the Netherlands; † National Institute for Public Health and the Environment, Bilthoven, the Netherlands; ‡ Wilhelmina Hospital, Nijmegen, the Netherlands; § Amphia Hospital, Breda, the Netherlands; ¶ VUmc Medical Center, Amsterdam, the Netherlands; 1 These authors contributed equally to this article.

**Keywords:** *Staphylococcus aureus*, methicillin resistance, MRSA, animal reservoir, pig, cattle, research

## Abstract

In 2003 in the Netherlands, a new methicillin-resistant *Staphylococcus aureus (*MRSA) strain emerged that could not be typed with *Sma*1 pulsed-field gel electrophoresis (NT-MRSA). The association of NT-MRSA in humans with a reservoir in animals was investigated. The frequency of NT-MRSA increased from 0% in 2002 to >21% after intensified surveillance was implemented in July 2006. Geographically, NT-MRSA clustered with pig farming. A case–control study showed that carriers of NT-MRSA were more often pig or cattle farmers (pig farmers odds ratio [OR] 12.2, 95% confidence interval [CI] 3.1–48.6; cattle farmers OR 19.7, 95% CI 2.3–169.5). Molecular typing showed that the NT-MRSA strains belonged to a new clonal complex, ST 398. This study shows that MRSA from an animal reservoir has recently entered the human population and is now responsible for >20% of all MRSA in the Netherlands.

## Abstract 2

MRSA from an animal reservoir has recently entered the human population and is now responsible for >20% of all MRSA in the Netherlands.

Methicillin-resistant *Staphylococcus aureus* (MRSA) has traditionally been considered a nosocomial pathogen. However, for several years the number of reports of so-called community-onset MRSA (CO-MRSA) has been rapidly increasing (*1*). CO-MRSA has no relation to healthcare and is usually associated with the presence of Panton-Valentine leukocidin toxin (PVL) and SCC*mec* types IV and V (*2*,*3*). In 2004 and 2005, some unexpected cases of MRSA were found in patients who were associated with pig farms (*4*,*5*). Genotyping showed that these MRSA isolates were nontypable by pulsed-field gel electrophoresis (PFGE) and belonged to 1 *spa* type (t108). The aims of this study were to determine if nontypable MRSA (NT-MRSA) isolates are associated with pig farming and to compare the phenotypic, genotypic, and epidemiologic features of NT-MRSA with those of typable MRSA strains.

## Methods

### National MRSA Database

The National Institute for Public Health and the Environment (RIVM) is the national reference center for MRSA in the Netherlands (www.rivm.nl/mrsa). According to national guidelines, all microbiology laboratories send the first isolate of newly identified carriers of MRSA to RIVM. Strains are confirmed to be MRSA by a Martineau PCR and by *mecA* PCR assay (*6*,*7*). Since 2002, all strains are typed by using PFGE (*8*), and the presence of PVL genes is determined (*9*).

### Selection of Cases and Controls

Cases and controls were selected from the national MRSA database at RIVM. Case-patients were defined as persons carrying NT-MRSA who provided the first isolate from a cluster of 1 particular referring laboratory (index-patient) in the period January 2003 to September 2005. Cases were considered to be secondary to an index case when the strain was isolated within 3 months after the previous isolate with the same PFGE typing result. Controls were persons who carried MRSA that was typable with PFGE and who also fulfilled the index-patient definition. Controls were derived from the laboratories that provided cases and were selected at random. Twice as many controls as case-patients were selected.

### Collection of Epidemiologic Background Information

Data were collected by questionnaires that were sent to the referring laboratories. The questionnaire contained items about patient characteristics (birth date, sex, postal code, presence or absence of infection, hospital admission dates, profession, profession of partner, profession of parents, and contact with animals, e.g., pigs, cows, horses, chickens, cats, or dogs) and microbiologic data (isolation date, source of culture, medical specialty). All data were collected and entered into the database without our knowing whether it concerned a case or control.

Initially, 41 cases and 82 controls from 26 different laboratories were selected from the national database. The response rate was 98% (40 cases and 81 controls). During workup, 5 cases and 5 controls were excluded for the following reasons: the confirmation test of the isolate indicated that it was not methicillin resistant (1 case), or the case did not fulfill the case definition because it was not the first case from a cluster (4 cases). Since 2 of these cases were from laboratories that had no other case in the study, the accompanying controls were excluded (n = 3). Two controls were identified outside the study period. Finally, 35 cases and 76 controls from 24 different laboratories were included in the analysis.

### Molecular Typing and Susceptibility Testing

All MRSA isolates were typed by PFGE (*8*). All isolates from case-patients and 74 isolates from controls were typed by *spa* typing (*10*). Multilocus sequence typing (MLST) was performed on all case isolates, as well as on 1 strain of each *spa* type of the control isolates (n = 37) (*11*). PCR of the staphylococcal chromosome cassette (SCC*mec*) was performed according to Zhang et al. on all isolates from case-patients and 74 isolates from controls (*12*). The presence or absence of PVL genes (*lukS*-*PV*/*lukF*-*PV*) was determined in all case isolates and in 71 control isolates. The PVL genes were detected by PCR according to the method of Lina et al. (*13*). The susceptibility to antimicrobial agents was tested for 32 case isolates and 74 control isolates, according to CLSI guidelines that used Mueller-Hinton agar and multipoint inoculation (*14*).

### Statistical Analysis

Data were entered into an Excel database (Microsoft Windows version 97 SR-2, Redmond, WA, USA) and further analyzed by using SAS (version 9.1) software package (SAS Institute Inc., Cary, NC, USA). Chi-square test and Fisher exact test for ordinal variables and Student *t* test for continuous variables were used for univariate analysis. Variables associated with both case-control status and the exposure (i.e., contact with pigs or cattle, respectively) with a p value <0.2 were included in the multivariate logistic regression model. If such variables changed the risk estimate for >10%, they were left in the model. All statistical tests were 2-sided, and a p value <0.05 was considered statistically significant.

## Results

### Epidemic Curve

The first NT-MRSA isolate was found in February 2003. In subsequent years, an increasing number of NT-MRSA isolates were found. The percentage of NT-MRSA relative to the total number of MRSA isolates in the Netherlands that were unique or first from a cluster rose from 0% in 2002 to 5.5% in the first half of 2006 and to >21% in the second half of 2006, after the introduction of intensified surveillance in July 2006.

### Geographic Distribution

**Figure 1:**
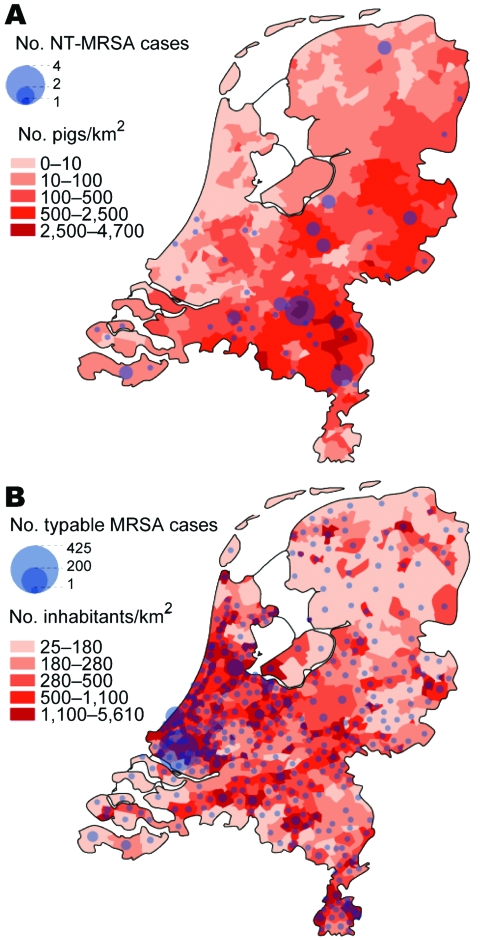
A) Number of nontypable methicillin-resistant *Staphylococcus aureus* (NT-MRSA) isolates per municipality received at the National Institute for Public Health and the Environment (RIVM), Bilthoven, the Netherlands, January 2003–June 2005. The background color represents the density of pigs per km^2^ in 2003. B) Number of typable MRSA per municipality received at the RIVM January 2003–June 2005. The background color represents the population density per km^2^ (source: CBS Statline).

Figure 1 shows the geographic distribution of NT-MRSA and typable MRSA isolates, plotted over the density of the pig and human populations, respectively. The density of NT-MRSA isolates corresponds to the density of pig farming, whereas the density of typable strains corresponds to the density of the human population. The density of cattle farms is more or less identical to the density of pig farms.

### Epidemiologic Data

Results of the univariate analysis are shown in Table 1. Comparable values were observed for the baseline characteristics of sex and age. Case-patients more often lived in rural areas and indicated more frequent contact with pigs or cattle than did controls. Controls were more often associated with healthcare facilities.

**Table 1:** Results of univariate analysis of case-control study, the Netherlands, February 2007*

Variable	Cases			Controls		Odds ratio (95% CI)†	p value
	No.	No. (%) or mean ± SD with variable		No.	No. (%) or mean ± SD with variable		
Gender (male)	35	20 (57)		76	36 (47)	1.5 (0.7–3.3)	0.34
Age, y	35	42.7 ± 25.3		76	47.3 ± 24.7		0.37
Residence	35			75			
Rural area		14 (40)			6 (8)	7.7 (2.6–22.7)§	<0.01
Urban area		20 (57)			66 (85)		
Foreign country		1 (3)			3 (4)		
Contact with pigs	29	11 (38)		63	3 (5)	12.2 (3.1–48.6)	<0.01
Contact with cattle	29	7 (24)		63	1 (2)	19.7 (2.3–169.5)	<0.01
Unexpected MRSA	35	27 (77)		76	34 (45)	4.2 (1.7–10.4)	<0.01
Probable source	35			76			
Healthcare		5 (14)			39 (51)		0.01
Foreign country		3 (9)			5 (7)		
Other		12 (34)			10 (13)		
Unknown		15 (43)			22 (29)		
Active infection	35	19 (54)		76	29 (38)	1.9 (0.9–4.3)	0.11
Skin/soft tissue		10 (56)			24 (83)	0.3 (0.1–1.0)	0.05
Airways		3 (17)			0		
Other		6 (28)			5 (17)		
Hospital admission	35	17 (49)		76	24 (32)	2.0 (0.9–4.6)	0.08
Hospital stay, d	16	18.9 ± 20.2		22	23.5 ± 30.9		0.60

*SD, standard deviation; CI, confidence interval; No., number of cases or controls for whom data are available. †Odds ratio was determined for rural area relative to urban area.

Among case-patients, MRSA was more frequently found in clinical samples (an unexpected finding) compared with controls, whose MRSA was found more often by targeted screening in nose, throat, and perineum. Among persons infected by MRSA, respiratory tract infections were more frequent in case-patients, whereas skin and soft tissue infections predominated in controls.

Multivariate analysis that used a model with the variables describing type of residence (rural vs. other) and contact with pigs, cattle, cats, and dogs (yes, no, or unknown) showed that contact with pigs and contact with cattle were independent statistically significant variables. The adjusted odds ratios (OR) for pigs and cattle were 9.4 (95% confidence interval [CI] 1.8–47.7) and 13.5 (95% CI 1.0–179.3), respectively.

### Molecular Typing

**Figure 2:**
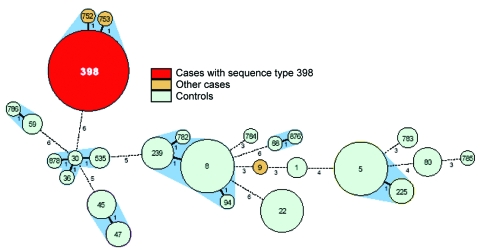
Genetic relatedness of methicillin-resistant *Staphylococcus aureus* from cases and controls, represented as a minimum spanning tree based on multilocus sequence typing (MLST) profiles. Each circle represents a sequence type, and numbers in the circles denote the sequence type. The size of the circle indicates the number of isolates with this sequence type. The number under and right of the lines connecting types denotes the number of differences in MLST profiles. The halos surrounding the circles indicate complexes of sequence types that differ by <3 loci.

Thirty-two of 35 case-patients had MLST sequence type (ST) 398; 1 had ST 9; and the remaining 2 had ST 752 and 753, closely related to 398 (Figure 2). Among case-patients, the most frequent *spa* types were t108, t011, and t034 (Table 2). These MLST and *spa* types were not found among the controls. Twenty-two different STs and 37 different *spa* types were found in the controls (Table 2 and Figure 2).

**Table 2:** Typing results for cases and the number of controls with the same type, the Netherlands, February 2007

Type	Cases, no. (%)	Controls, no. (%)	p value
*spa*			
t108	14 (40)	0	<0.01
t011	8 (23)	0	
t034	6 (17)	0	
t571	3 (9)	0	
t567	2 (6)	0	
t337	1 (3)	0	
t898	1 (3)	0	
SCC*mec*			
I	0	4 (9)	<0.01
II	0	7 (16)	
III	4 (17)	6 (14)	
IV	2 (8)	21 (49)	
V	18 (75)	5 (12)	

Panton-Valentine leukocidin

3 (9)

10 (14)

0.21

SCC*mec* typing showed that in isolates from cases SCC*mec* types III, IV, and V were found, whereas in isolates from controls all SCC*mec* types were found (Table 2). For 11 cases and 33 controls, the SCC*mec* type could not be determined. There was no difference in the presence of the PVL genes (Table 2).

### Antimicrobial Agent Susceptibility

Table 3 shows the percentage of strains that were resistant to various antimicrobial agents. Isolates from case-patients were significantly more often resistant to doxycycline and clindamycin than were isolates from controls.

**Table 3:** Number and percentage of resistant MRSA isolates for various antimicrobial agents, the Netherlands, February 2007*

Agent	Cases, no. (%)	Controls, no. (%)	p value
Doxycycline	25 (78)	10 (14)	<0.01
Ciprofloxacin	1 (3)	36 (49)	<0.01
Tobramycin	4 (13)	25 (34)	0.02
Gentamicin	2 (6)	12(16)	0.14
Clindamycin	12 (38)	15 (20)	0.05
Erythromycin	15 (46)	29 (39)	0.35
Cotrimoxazole	0	7 (10)	0.07
Rifampin	0	6 (8)	0.11
Mupirocin	0	5 (7)	0.15
Vancomycin	0	0	

*MRSA, methicillin-resistant *Staphylococcus aureus.*

## Discussion

A new type of MRSA recently emerged in the Netherlands. The first isolate was found in 2003, and since then it has been found with increasing frequency. The geographic origin of NT-MRSA correlates with the density of pig populations. This association was confirmed by the results from this case-control study, which show that NT-MRSA is significantly related to contact with pigs. In addition, a significant association was found with cattle. After multivariate analysis, contact with pigs and cattle were the only 2 significant independent variables. Screening of a representative sample of pigs in the Netherlands was recently performed and showed that nearly 40% of the pigs were colonized with a comparable strain of MRSA (MLST 398) and that ≈80% of the pig farms were affected (*15*). The association between NT-MRSA and cattle was not expected when this study was initiated and needs further evaluation.

On the basis of the above-mentioned findings, we conclude that this new MRSA strain is of animal origin (pigs and probably cows). Transmission of MRSA between animals and humans has previously been described, e.g., associated with colonized companion animals, horses, and persons who take care of them (*16*–*19*). However, the MRSA clones in these reports were known human clones, suggesting human-to-animal transmission in origin. Baptiste et al. found specific PFGE clones in horses that were never observed before (*20*). Until now, transmission of these clones to humans has not been reported.

We assume that this problem is not limited to the Netherlands. First, widespread dissemination in pigs in the Netherlands has been found. When one considers the intensive international transport of pigs, it is unlikely that this situation is limited to the Netherlands. Second, 3 of the case-patients came from abroad, 1 tourist and 2 adopted children from Asia. Also, MLST 398 was recently found in animals (pig, dog, and foal) and in humans in Germany (*21*). Finally, in Hong Kong Special Administrative Region, People’s Republic of China, MRSA with MLST 398 has been found in 2 patients with bacteremia (*22*).

The origin of the current NT-MRSA situation is difficult to elucidate. One earlier study can be found on carriage of *S. aureus* in pig farmers and pigs in France (*23*). It reported an increased carriage rate in pig farmers caused by transmission of *S. aureus* from pigs that also carried MLST ST 9 and 398. Further typing of the French ST 398 isolates at RIVM showed homology with the Dutch isolates. However, in the French study most of the MLST 398 strains were susceptible to β-lactam antimicrobial agents. The most likely explanation for the current findings is that MLST 398 is a commensal strain in pigs, which originally was methicillin susceptible. As most NT-MRSA isolates were resistant to doxycycline, the spread is facilitated by the abundant use of tetracyclines in pig and cattle farming (*15*).

What are the implications of these findings? Persons working or living in close contact with pigs or cows are at increased risk of becoming colonized and infected with MRSA. Infections can be severe, as is indicated by the hospital admission rate. Also, a case of endocarditis has been reported recently (*24*). At present, whether this strain is spreading further in the community is not clear. Before final recommendations for control can be made, the current size of the reservoir in farm animals and in humans has to be determined at an international level.
